# Identification and Characterization of Abiotic Stress–Responsive NF-YB Family Genes in *Medicago*

**DOI:** 10.3390/ijms23136906

**Published:** 2022-06-21

**Authors:** Wenxuan Du, Junfeng Yang, Qian Li, Chunfeng He, Yongzhen Pang

**Affiliations:** 1 Institute of Animal Science, Chinese Academy of Agricultural Sciences, Beijing 100193, China; n053727@163.com (W.D.); yangjf63@sina.com (J.Y.); qianli20210715@163.com (Q.L.); 82101215080@caas.cn (C.H.); 2 College of Horticulture, Hunan Agricultural University, Changsha 410128, China; 3 West Arid Region Grassland Resource and Ecology Key Laboratory, College of Grassland and Environmental Sciences, Xinjiang Agricultural University, Urumqi 830052, China

**Keywords:** *Medicago truncatula*, *Medicago sativa*, *NF-YB* genes, abiotic stresses, expression profiling

## Abstract

Nuclear factor YB (NF-YB) are plant-specific transcription factors that play a critical regulatory role in plant growth and development as well as in plant resistance against various stresses. In this study, a total of 49 *NF-YB* genes were identified from the genomes of *Medicago truncatula* and *Medicago sativa*. Multiple sequence alignment analysis showed that all of these NF-YB members contain DNA binding domain, NF-YA interaction domain and NF-YC interaction domain. Phylogenetic analysis suggested that these NF-YB proteins could be classified into five distinct clusters. We also analyzed the exon–intron organizations and conserved motifs of these *NF-YB* genes and their deduced proteins. We also found many stress-related *cis*-acting elements in their promoter region. In addition, analyses on genechip for *M. truncatula* and transcriptome data for *M. sativa* indicated that these *NF-YB* genes exhibited a distinct expression pattern in various tissues; many of these could be induced by drought and/or salt treatments. In particular, RT-qPCR analysis revealed that the expression levels of gene pairs *MsNF-YB27/MtNF-YB15* and *MsNF-YB28/MtNF-YB16* were significantly up-regulated under NaCl and mannitol treatments, indicating that they are most likely involved in salt and drought stress response. Taken together, our study on *NF-YB* family genes in *Medicago* is valuable for their functional characterization, as well as for the application of *NF-YB* genes in genetic breeding for high-yield and high-resistance alfalfa.

## 1. Introduction

Nuclear factor Y (NF-Y), also called heme-activated protein (HAP) or CCAAT binding factor (CBF), can be found in almost all eukaryotes [1,2]. The NF-Y Transcription Factors binds to *cis*-elements with the conserved core sequence CCAAT to activate or inhibit the expression of related functional genes in metabolism [3]. The NF-Y family protein consists of three different subunits: NF-YA, NF-YB, and NF-YC [4]. These three subunits are distinguished by conserved structure and protein length, with NF-YAs being longer than NF-YB and NF-YC. In general, NF-YA contains two structural domains, A1 and A2 [5], while the protein structures of NF-YB and NF-YC are similar to H2B and H2A histones, respectively [5]. Subunits NF-YB and NF-YC form dimers in the cytoplasm and then bind to the NF-YA protein and form a trimer in the nucleus [6]. Interestingly, in yeast and mammals, each NF-Y subunit is encoded by a single gene; but in plants, it is encoded by multiple genes, and the number of genes encoding individual subunits are also different from species to species [1].

Recent studies have shown that individual NF-Y subunits in plants are involved in many important growth processes, especially in embryogenesis [7], seed maturation [8], chloroplast synthesis [9], and tissue division [10]. It is worth noting that a large body of evidence has shown that *NF-YB* genes have multiple functions. For example, *AtNF-YB2* promotes the flowering process by increasing the expression of the key flowering genes Flowering locus T (FT) and Suppressor of Over-expression of Constans 1 (SOC1) [11]. *AtNF-YB2* and *AtNF-YB3* can interact with *AtNF-YC3*, *4*, and *9*, which play a crucial role in controlling flowering time via the photoperiod pathway [12]. In contrast to *AtNF-YB2*, other *NF-YB* genes in *A. thaliana* have been shown to function in drought tolerance, abscisic acid signaling transduction, embryo-like structures, and root elongation [13,14,15]. Typically, *AtNF-YB1* enhanced drought tolerance, neither in an ABA-dependent manner nor in an ABA-independent manner [13]. Over-expression of *AtNF-YB9* in transgenic *Arabidopsis* activates embryo-specific gene expression and leads to the formation of embryo-like structures in leaves, suggesting a role for *AtNF-YB9* in regulating embryonic development [16]. Aside from *A. thaliana*, the functional characterization of *NF-YB* genes has also been performed in several other plants species and has shown various biological roles. For example, *PdNF-YB7* from poplar increased drought tolerance through up-regulating downstream genes of the ABA-dependent pathway in *Arabidopsis* [17]. Moreover, *SiNF-YB8* can enhance drought and osmotic tolerance in tobacco [18]. Over-expression of *PwNF-YB3* from foxtail millet can significantly improve the tolerance of seedlings under salinity, drought, and osmotic stress [19]. These results demonstrated NF-YB genes from plants not only affect plant development but are also associated with plant resistance to abiotic stresses.

Alfalfa (*Medicago sativa* L.) is a perennial herb in the legume family with an available genome sequence [20]; it known for its high crude protein content and extensive primary root system, making it the most widely distributed cultivated forage grass in the world [21]. However, drought and high-salt soils are the two most important environmental factors that limit the growth of plants; therefore, mining key genes to improve alfalfa resistance to adversity has become one of the most effective approaches in solving this problem [22]. Previous studies have shown that the *NF-YB* subfamily plays key role in improving drought tolerance in plants such as *Arabidopsis* [13], tobacco [18], and maize [19]. Gene families such as *P450* [23], *HD-ZIP* [24], *SRS* [25], and *PcG* [26] in *Medicago* have been identified. These families are related to abiotic stress and growth, but the *NF-YB* gene family has not yet been reported. In light of this, we identified the *NF-YB* gene family from *Medicago truncatula* and *M. sativa* using genome-wide analysis. In total, 49 members of the *NF-YB* gene family were identified and further characterized, including information on sequence alignment, phylogenetic relationships, chromosome locations, gene structures, conserved motifs, and *cis*-element of the promoter region. In addition, genechip data for *M. truncatula* and transcriptome data for *M. sativa* were analyzed in the expression pattern of *NF-YB* genes, aiming to search for potential common response genes to drought and salt stress. Moreover, expression analysis under drought and salt stress treatments indicated that some *NF-YB* genes respond to drought and salt stress, and these genes can serve as candidates for genetic breeding of high resistance in *Medicago*.

## 2. Results

### 2.1. Identification of NF-YB Family Genes in Medicago

The conserved mode of NF-YB and the NF-Y proteins from *A. thaliana* and *O. sativa* were used as queries to identify *NF*-*YB* genes in *M. truncatula* and *M. sativa*. In total, 21 *MtNF-YB* and 28 *MsNF*-*YB* genes were identified and designated according to their location on chromosomes, as shown in Table 1. Previously, 24 *MtNF-YB* members were deposited in PlantTFDB with V4 genome sequencing results, and three of them were found to be duplicate sequences with the V5 genome sequencing data in the present study. Therefore, these 21 *MtNF-YB* were named according to their positions on the chromosome and were listed along with the corresponding gene locus deposited in PlantTFDB in Table 1. *MtNF-YB* and *MsNF-YB* genes encode proteins ranging from 91 to 257 and 78 to 289 amino acids in length, respectively. The detailed information of NF-YB family genes in *Medicago*, including sequence ID, chromosome location, amino acid length (aa), protein isoelectric point (PI) value and protein molecular weight (MW) (kDa) are listed in Table 1. Moreover, the corresponding *NF-YB* homologous genes of *M. truncatula* or *M. sativa* were identified based on sequence alignment. Subcellular location analysis showed that NF-YB proteins from *M. truncatula* and *M. sativa* were predicated to be located in nuclear (20 out of 21 *MtNF-YB*; 22 out of 28 *MsNF-YB*) or extracellular areas. Recent studies demonstrated that miRNAs located in the nucleus could act on CREs (e.g., promoters and enhancers) to regulate gene expression (activation or suppression) [27]. Those localized outside the cell may be closely related to cellular signal reception and transduction [28].

### 2.2. Multiple Sequence Alignment of NF-YB Genes in Medicago

**Figure 1:**
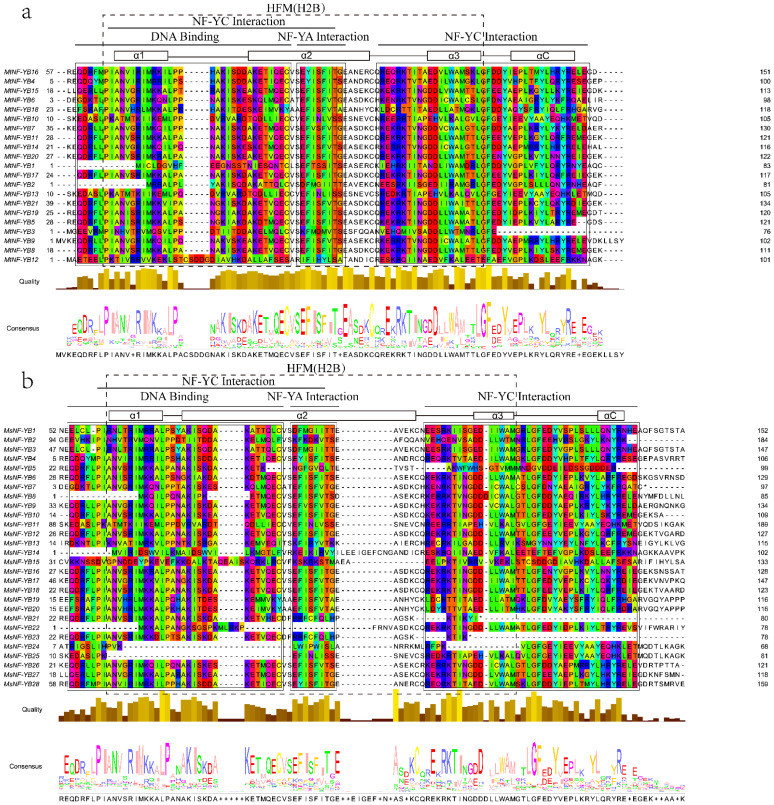
Multiple sequence alignment of MtNF-YBs and MsNF-YBs. The figure only showed partial sequences containing α1, α2, α3, and αC domains. The alignment was constructed by MEGA-X and visualized by Jalview. Residues with more than 50% similarity are shaded. Conserved regions (DNA binding, NF-YA interaction, and NF-YC interaction) are indicated at the top. (**a**) Multiple sequence alignment of NF-YB from *M. truncatula*. (**b**) Multiple sequence alignment of NF-YB from *M.* *sativa*.

To further investigate the conserved regions of NF-YB subfamilies in *Medicago*, multiple protein sequence alignments were analyzed using MEGA-X, and displayed via jalview (Figure 1). The results illustrated that the core regions for MtNF-YB and MsNF-YB were 92 and 88 amino acids in length, respectively, which were close to the average length reported previously [29]. In addition, most NF-YBs from *M. truncatula* and *M. sativa* also contained DNA binding α1, NF-YA interaction α2, and NF-YC interaction α3/αC, which have a similar structure as the histone-fold motif (HFM) of the core histone H2B [30]. Among them, MtNF-YB3 lacks the αC domain, MsNF-YB21 and 23 lack α3 and αC domains, MsNF-YB25 lacks the α1 domain, and MsNF-YB24 lacks α1 and α3 domains, which may be due to the genome sequences.

### 2.3. Phylogenetic Analysis of NF-YB Proteins

**Figure 2:**
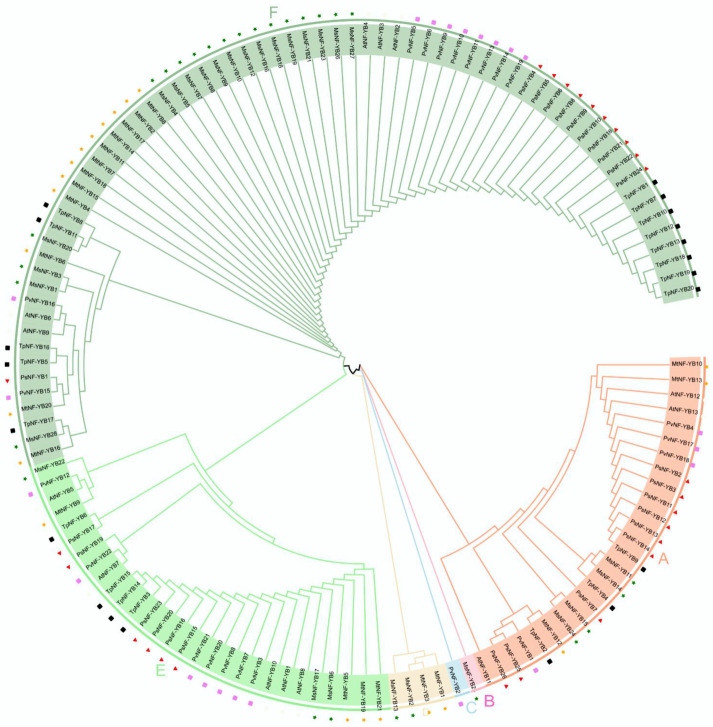
Phylogenetic analysis of NF-YB proteins from *M. truncatula*, *M. sativa*, *Arabidopsis*, *P. vulgaris*, *P. sativum*, and *T. pratense*. Full-length protein sequences of NF-YBs were used to construct the tree by using MEGA-X based on the Neighbor-Joining (NJ) method with a bootstrap value of 1000 replicates. Subfamilies are highlighted with different colors. The green solid pentagrams, orange solid pentagrams, hollow circles, violent diamonds, red triangles, and blank squares represent NF-YB proteins from *M. truncatula* (Mt), *M. sativa* (Ms), *A. thaliana* (At), *P. vulgaris* (Pv), *P. sativum* (Ps), and *T. pretense* (Tp), respectively.

To better understand the evolutionary relationships of the *NF*-*YB* gene, a neighbor-joining (NJ) phylogenetic tree was constructed with the NF-YB proteins from *M. truncatula*, *M. sativa*, *A. thaliana*, *P. vulgaris*, *P. sativum*, and *T. pratense*. The details are shown in Table S2. The NF-YB proteins were divided into six clades, designated as A, B, C, D, E, and F. The F clade was the largest group, containing 69 NF-YB proteins, whereas the B and C clade were the smallest, consisting of only one member (only MsNF-YB25 in group B and PcNF-YB2 in group C), indicating that NF-YB proteins were distributed unevenly in different clades (Figure 2). Among them, three AtNF-YB members were found in each of clusters A and E. Notably, only two members from *M. sativa* (MsNF-YB2, 13) and *M. truncatula* (MtNF-YB2, 13) were present in cluster D, demonstrating that these genes may play specific roles in the evolutionary process (Figure 2).

### 2.4. Analysis of Gene Structure and Conserved Motifs of NF-YB Genes in Medicago

**Figure 3:**
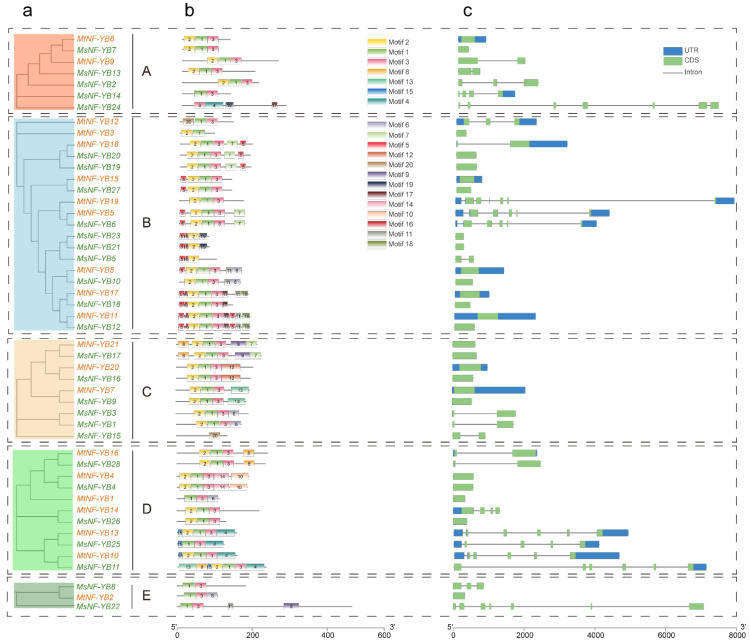
Phylogenetic relationships, motifs, and gene structure of *NF-YB* genes from *M. truncatula* and *M. sativa* (**a**–**c**). The groups and color of the phylogenetic tree are the same as in Figure 2. The motifs are indicated in different colored boxes with different numbers, and sequence information for each motif is provided in Supplementary Figure S1. Blue boxes indicate 5′- and 3′- untranslated regions; green boxes indicate exons; black lines indicate introns.

To comprehensively study the function of *NF-YB* genes, we performed analysis on gene structure and conserved motifs. We performed another phylogenetic analyses for NF-YB from *M. sativa* and *M. truncatula* and found they were regrouped into five clusters. As shown in Figure 3a, all NF-YB proteins were classified into five clades that were consistent with the phylogenetic relationships, as illustrated in Figure 2. The MEME analysis tool was used to predict conserved motifs in *NF-YB* genes (Figure 3b). A total of 20 motifs were identified; we found that all 21 MtNF-YB members contained motif 1. In total, 23 out of 28 MsNF-YB members contained motif 1, except for MsNF-YB24, 23, 21, 5, and 15. In addition, most NF-YB contained similar motifs, for example, motifs 2 and 3 were widely distributed in most NF-YB. We also found that NF-YB proteins with close phylogenetic relationships exhibited similar motif arrangements, for example, motifs 5, 11, 16, 17, and 18 only present in cluster B. The specificity of these motifs may result in functional differences among NF-YB within each cluster. To elucidate the gene structure of *NF-YB* family genes, we compared the coding sequences with their corresponding genomic sequences to determine the positions of exons and introns. As shown in Figure 3c, the numbers of exons ranged from one to seven, where genes with one exon accounted for 53% of the total *NF-YB* genes, most of which were from B and C clades. These results indicate that the intron/exon distribution in *NF-YB* genes from *Medicago* is highly variable. In addition, 29 genes lacked one or two of the 5′- or 3′- untranslated region (UTR), particularly in cluster E, which may be due to the genome annotation.

### 2.5. Analyses of Chromosomal Distribution and Synteny of NF-YB Genes in Medicago

**Figure 4:**
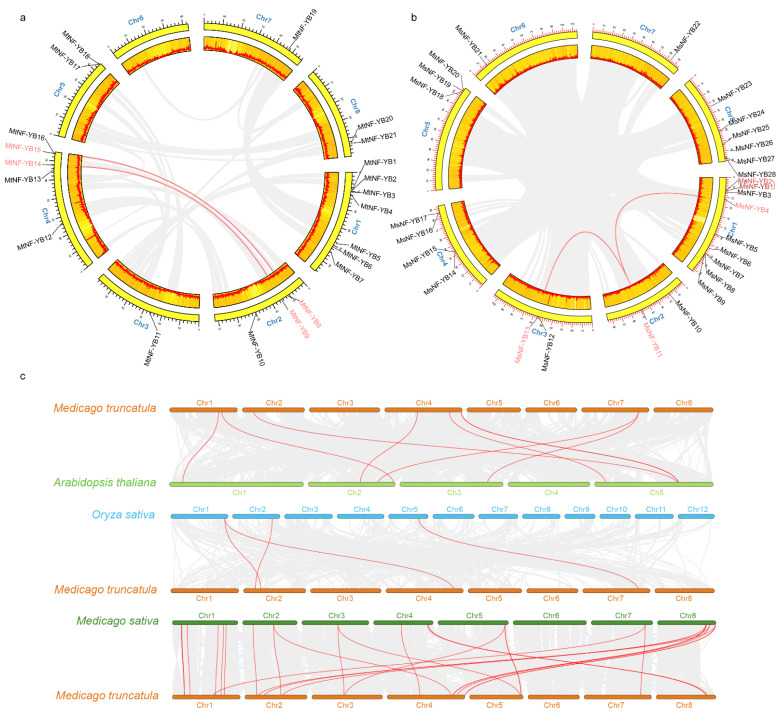
Chromosome distributions of NF-YBs in *M. truncatula* and *M. sativa*. The chromosomal location and interchromosomal relationship of *M. truncatula* (**a**) and *M. sativa* (**b**). The segmentally duplicated and tandem duplicated genes are connected by red curves. (**c**) Synteny analysis of NF-YB genes between *A. thaliana* and *M. truncatula*, *O. sativa* and *M. truncatula*, *M. sativa* and *M. truncatula*. Gray lines in background indicate the collinear blocks between *M. truncatula* and *A. thaliana*/*O. sativa*/*M. sativa*, and red lines highlight syntenic NF-YB gene pairs.

All *NF-YB* genes were unevenly distributed on chromosomes in *M. truncatula* and *M. sativa* (Figure 4a,b). The *NF-YB* genes of *M. truncatula* were widely distributed on chr1 with seven members, followed by chr4 with five members (Figure 4a). Notably, chr6 had no *NF-YB* (Figure 4a). Nine *NF-YB* genes of *M. sativa* were also distributed on chr1, and one to six genes were distributed on chr2-6 (Figure 4b).

Tandem duplication, segmental duplication, and whole-genome duplication are the main impetus for gene family expansion [31]. Two pairs of segmental duplication were found in *M. truncatula* (*MtNF-YB8*/*MtNF-YB15* and *MtNF-YB9*/*MtNF-YB14*) and *M. sativa* (*MsNF-YB4*/*MsNF-YB11* and *MsNF-YB11*/*MsNF-YB13*), respectively (Figure 4a,b and Supplementary file S1). Notably, both segmental duplications in *M. sativa* were homologous to *MsNF-YB11*, suggesting that this gene may play a unique function in the evolutionary process. In addition, only one pair of tandem repeat events (*MsNF-YB1*/*MsNF-YB2*) was found in *M. sativa* (Figure 4b and Supplementary file S1), while this event was absent in *M. truncatula* (Figure 4a).

Furthermore, three comparative syntenic maps of *A. thaliana*, *O. sativa,* and *M. sativa* separately associated with *M*. *truncatula* were constructed to illustrate the evolutionary relationship of the *NF-YB* gene family (Figure 4c). Unsurprisingly, *M*. *truncatula* and *M*. *sativa* had the most collinear gene pairs, with 24 pairs, while *M*. *truncatula* and *O. sativa* had the least number of collinear gene pairs, with only four pairs. Moreover, *M*. *truncatula* and the representative plant species *Arabidopsis* had eight collinear gene pairs (Supplementary file S1). Notably, *MtNF-YB8* and *MtNF-YB19* had synteny relationships with each of the other three species, which may play a special role in the growth and development of *M*. *truncatula*.

Over the course of evolutionary history, duplicated genes have three potential evolutionary fates: non-functionalization, neo-functionalization, and sub-functionalization [32]. In comparing the non-synonymous (Ka) and synonymous substitution (Ks) rates of substitution (Ka/Ks), one could infer the magnitude of selective constraint and positive selection. Generally, Ka/Ks > 1, Ka/Ks = 1, and Ka/Ks < 1 indicate positive selection, neutral evolution, and purifying selection, respectively [33]. In the present study, the Ka, Ks, and Ka/Ks of NF-YB homologous gene pairs were estimated in *Medicago* (Supplementary file S1). This showed that the *MsNF-YB1*/*MtNF-YB2* homologous gene pairs had Ka/Ks ratios of 1.821, indicating a high degree of positive selection. In sharp contrast to this gene pair, we found that the Ka/Ks ratios of other *NF-YB* gene homologous pairs were less than 0.5, and that the ratios of another five homologous pairs were even smaller than 0.1, suggesting that *NF-YB* genes have undergone purifying selection after segmental and whole-genome duplications.

### 2.6. Analysis of Cis-Elements in the Promoter Sequences of NF-YB Genes in Medicago

**Figure 5:**
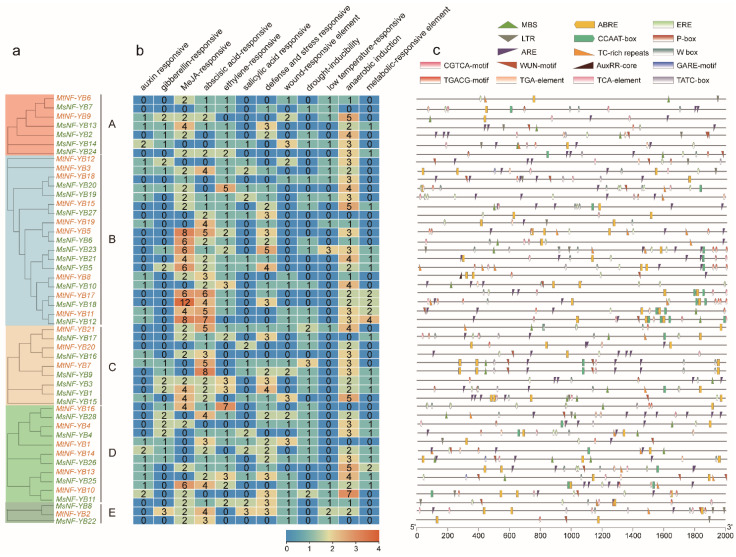
Putative *cis*-elements and transcription factor binding sites in the promoter regions of *NF-YB* genes from *M. truncatula* and *M. sativa*. (**a**) The groups and color are as indicated in Figure 2. (**b**) The color and number of the grid indicate numbers of different *cis*-acting elements in these *NF-YB* genes. (**c**) The colored blocks represent different types of *cis*-acting elements and their locations in each *NF-YB* gene.

The *cis*-acting element is important for the binding of transcription factors to control the expression of their target genes. In this study, we focused on hormones and abiotic stress related *cis*-acting elements, including auxin-responsive (AuxRE-core), gibberellin-responsive (GARE-motif, P-box, TATC-box), MeJA-responsive (TGACG-motif, CGTCA-motif), abscisic acid–responsive (ABRE), ethylene-responsive (ERE), salicylic acid–responsive (TCA-element), defense- and stress-responsive (TC-rich repeats, W-box), wound-responsive (WUN motif), MYB binding site involved in drought-inducibility (MBS), low temperature–responsive (LTR), anaerobic induction (ARE) and metabolic-responsive (CCAAT-box) (Figure 5 and Supplementary file S2).

Overall, the promoters of *NF-YB* genes contained various *cis*-acting elements with different numbers. In particular, almost all *NF-YB* genes contain a high number of ARE elements (*p* < 0.05 for 11 of them with Fisher’s exact test), and they may play a crucial role in anaerobic induction response in roots of *Medicago*. Methyl jasmonate (MeJA), as a wounding-related phytohormone, is able to stimulate the expression of defense-like genes [34]. Interestingly, *NF-YB* genes with relatively more MeJA-responsive elements were grouped in cluster B (Figure 5b,c), with 12 in *MsNF-YB18* (*p* < 0.01 with Fisher’s exact test), indicating that *NF-YB* genes in this cluster play a specific role in resistance against wounding stress. It is generally known that three *cis*-elements, ABRE, MBS, and W-box, are related with responsiveness to drought-induced signaling and regulation of downstream gene expression [35]. Our results showed that many *NF-YB* genes contained more ABRE and W-box elements (*p* < 0.05 for nearly half of them with Fisher’s exact test), while MBS elements were grouped in C and D, indicating that the *NF-YB* family gene plays a role in drought resistance in *Medicago*. Notably, the NF-Y family protein binds specifically to the CCAAT *cis*-acting elements, though it is not widely distributed in the *NF-YB* family of *Medicago* (Figure 5b,c).

### 2.7. Expression Patterns of NF-YB Genes in Different Tissues

**Figure 6:**
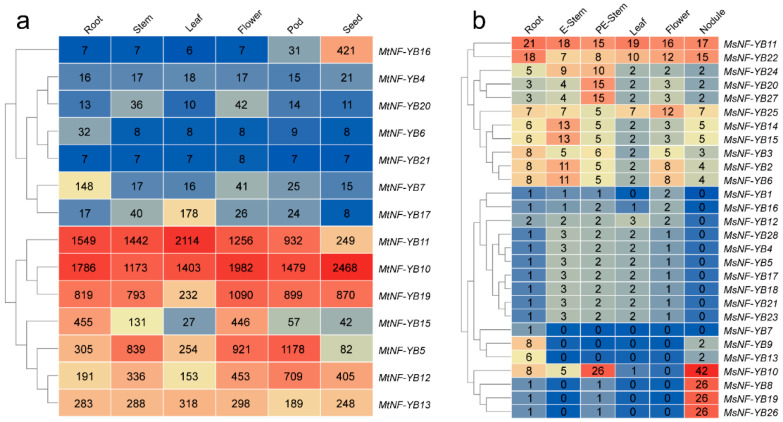
Expression profiles of *NF-YB* genes in different tissues of *Medicago*. (**a**) Expression profiles with the log2(FPKM) values of *MtNF-YB* genes in different tissues retrieved from the genechip dataset. (**b**) Expression profiles with the log2 (FPKM) values of *MsNF-YB* genes in different tissues retrieved from transcriptome data. Red represents high expression and blue represents low expression.

We investigated the expression patterns of *NF-YBs* in various tissues of *M. truncatula* with the genechip dataset from the MtGEA web server, including roots, stems, leaves, flowers, pods, and seeds (Figure 6a). Overall, half (7) of the 14 *MtNF-YB* genes were expressed at a relatively low level in these tissues, and the other half were expressed at a relatively high level (Figure 6a). Among the lowly expressed seven genes, *MtNF-YB16* was highly expressed in seeds, *MtNF-YB7* in roots, and *MtNF-YB17* in leaves (Figure 6a). Among the highly expressed seven genes, *MtNF-YB5* and *11* were lowly expressed in seeds, and *MtNF-YB15* and *19* in leaves (Figure 6a).

For *M. sativa*, gene expression levels in six tissues were analyzed based on transcriptome data, including roots, elongated stems, pre-elongated stems, leaves, flowers, and nodules (Figure 6b). The transcriptome data for *MsNF-YBs* could be divided into two distinct categories: higher expression genes (from *MsNF-YB11* to *MsNF-YB6*, Figure 6b from top to bottom) and lower expression genes (from *MsNF-YB1* to *MsNF-YB26*, Figure 6b from top to bottom). Compared with other genes, *MsNF-YB11* and *MsNF-YB22* were highly expressed in all tissues (Figure 6b). Interestingly, the expression of four genes (*MsNF-YB10*, *8*, *19*, *26*) in nodules was significantly higher than in other tissues or than the remaining *MsNF-YB* genes, and these genes may play a specific role in nodules.

### 2.8. Expression Levels of NF-YB Genes in Medicago under Stress

**Figure 7:**
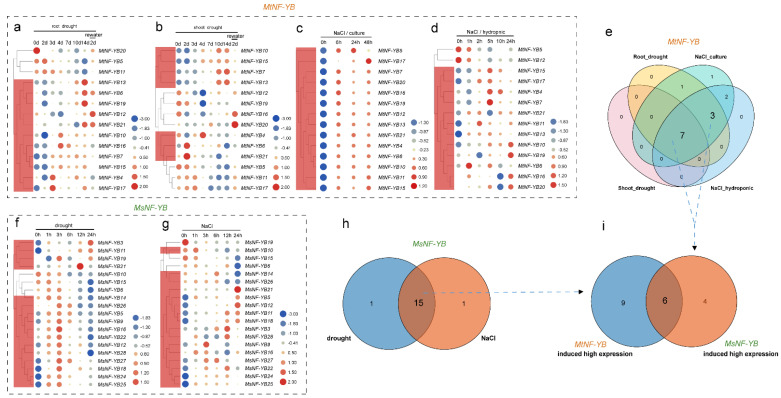
Genechip data of *MtNF-YB,* transcriptome data of *MsNF-YB,* and gene expression profiles of different treatments and different times. Each column represents a treatment, each row represents a gene, and each member is normalized in the same column. The relative expression levels are log2-transformed and visualized for heatmap. The color of the circles from blue to red shows the expression level from negative to positive values after normalization. The size of the circles represents the amount of gene expression. The red box indicates genes with increased expression. (**a**,**b**) Expression level of *MtNF-YB* genes in roots (**a**) or shoots (**b**) under different drought treatment times. (**c**,**d**) Expression levels of *MtNF-YB* genes in roots under different treatment times for NaCl-culture (**c**) or NaCl-hydroponic time (**d**). (**e**) Venn diagram of gene expression levels of *MtNF-YB* genes after four different treatments. (**f**) Expression levels of *MsNF-YB* genes under different drought treatment time. (**g**) Expression levels of *MsNF-YB* genes under NaCl treatment at different time points. (**h**) Venn diagram of expression levels of *MsNF-YB* genes after two different treatments. (**i**) Venn diagram of genes whose expression levels were increased by stress treatment; these genes were selected based on the homolog listed in Table 1.

It was reported that *NF-YB* genes are involved in a number of important processes, such as embryo and seed development, flowering, photosynthesis, and stress responses [36]. Therefore, we also analyzed their expression level with genechip data for *M. truncatula,* including roots under in vitro culture salinity and hydroponic salinity, as well as roots and shoots under drought treatment. One probe set was selected as representative for each *MtNF-YB* gene, and 14 out of 21 *MtNF-YB* genes had their corresponding probe set (Figure 7a and Supplementary file S3).

Meanwhile, transcriptome data of *M. sativa* for samples from roots under drought and NaCl treatment were also analyzed (Supplementary file S4). Due to extremely low expression of some genes, only 19 *MsNF-YB* genes were presented in Figure 7f,g. To further screen stress-responsive genes under different stress treatments, the average change folds of each gene under individual treatment (total change folds for all treatments/number of treatments) of *MtNF-YB* and *MsNF*-*YB* genes were calculated (Figure 7, Supplementary files S3 and S4).

In *M. truncatula*, 11 genes (*MtNF-YB13*, *MtNF*-*YB6*, *MtNF*-*YB19*, etc.) and 7 genes (*MtNF*-*YB10*, *MtNF*-*YB15*, *MtNF*-*YB7*, etc.) were highly induced in roots or in shoots under drought treatment, respectively (Figure 7a,b). In contrast, all genes were significantly up-regulated at different levels under NaCl treatment, except *MtNF-YB5* and *MtNF*-*YB12* genes, which were down-regulated under hydroponic NaCl treatment (Figure 7c,d). Taken together, as shown in the Venn diagram, ten genes were highly expressed under at least three stress treatments (Figure 7e).

In *M. sativa*, eleven *NF-YB* genes (*MsNF-YB3*, *MsNF*-*YB11*, *MsNF*-*YB19*, etc.) were highly induced under drought treatment (Figure 7f), and 11 genes (*MsNF-YB10*, *MsNF-YB14*, *MsNF-YB26*, etc.) were highly induced under NaCl treatment with different fold changes (Figure 7g). The Venn diagram showed that 15 genes were highly expressed under both NaCl and drought treatment (Figure 7h).

Homologous genes of 15 NaCl and drought responsive *MsNF-YB* genes were identified from *M. sativa* (Figure 7h and Table S3), and they were further compared with the 10 *MtNF*-*YB* genes that were screened from Figure 7e. It was revealed that six of them were common genes, as shown in the Venn diagram (Figure 7i and Table S3). Therefore, these six homologous gene pairs from both *M. truncatula* and *M. sativa* (*MtNF-YB7*/*MsNF-YB9*, *MtNF-YB10*/*MsNF-YB11*, *MtNF-YB13*/*MsNF-YB11*, *MtNF-YB15*/*MsNF-YB27*, *MtNF-YB16*/*MsNF-YB28,* and *MtNF-YB17*/*MsNF-YB28*) were selected as candidate genes for subsequent investigation.

### 2.9. Validation of the Expression Profile of Stress-Responsive NF-YB Genes by RT-qPCR

**Figure 8:**
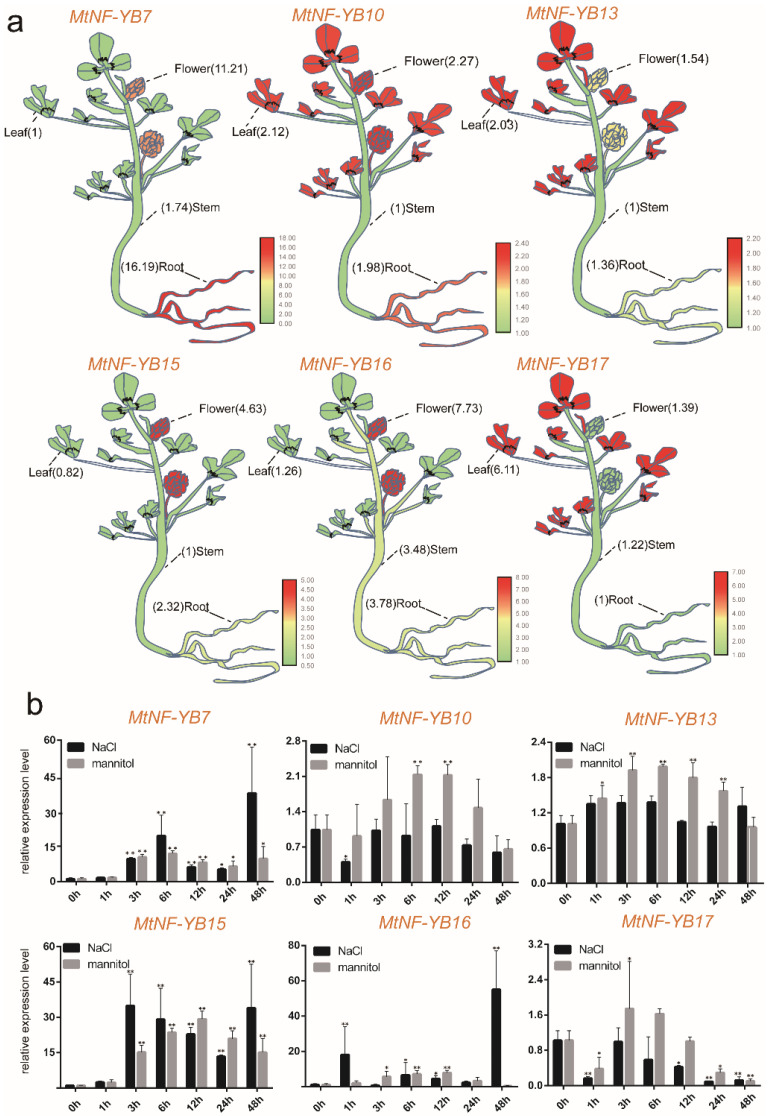
Quantification of gene expression levels of selected *NF-YB* genes from *M. truncatula* using RT-qPCR. (**a**) Expression level of *MtNF-YB* genes in different tissues. (**b**) Expression level of *MtNF-YB* genes under NaCl and mannitol stress. Data are average of three independent biological samples ± SE, and vertical bars indicate standard deviation. ** indicates *p* < 0.01, and * indicates *p* < 0.05.

In order to verify the expression profiles of *NF-YB* genes from genechip for *M. truncatula* and transcriptome data for *M. sativa*, RT-qPCR was carried out for the verification of six candidate gene pairs. The expression levels of these above-mentioned six genes in four tissues (roots, stems, leaves, and flowers) were further verified by RT-qPCR, with their lowest expression as a control, as shown in the model figure (Figure 8a and Supplementary file S5). Notably, the expression profile of almost all of these genes was consistent with genechip data (Figure 6a and Figure 8a); for example, *MtNF-YB7*, *MtNF-YB10,* and *MtNF-YB17* showed the highest expression in roots, leaves, and leaves, respectively. As an exception, *MtNF-YB16* was expressed with a significantly higher level in flowers than in other tissues, whereas it was expressed at a similar level to the genechip data in the four tissues (Figure 6a and Figure 8a).

Moreover, seedlings were treated with NaCl or mannitol at 1 h, 3 h, 6 h, 12 h, 24 h, 48 h and used for RT-qPCR analysis. RT-qPCR showed that *MtNF-YB7* and *MtNF-YB15* were highly induced by both NaCl and mannitol treatment from 3 h to 48 h (Figure 8b and Supplementary file S5). The expression level of *MtNF-YB10* was induced by mannitol at 6 h and 12 h, and *MtNF-YB13* at 1 h, 3 h, 6 h, 12 h, and 24 h, though the fold change was not more than 2.5-fold (Figure 8b). The expression level of *MtNF-YB16* was induced by more than 15-fold and 50-fold at 1 h and 48 h by NaCl treatment (Figure 8b). However, the expression of *MtNF-YB17* appeared to be repressed by both treatments, except for a slight increase by the mannitol treatment at 3 h (Figure 8b).

**Figure 9:**
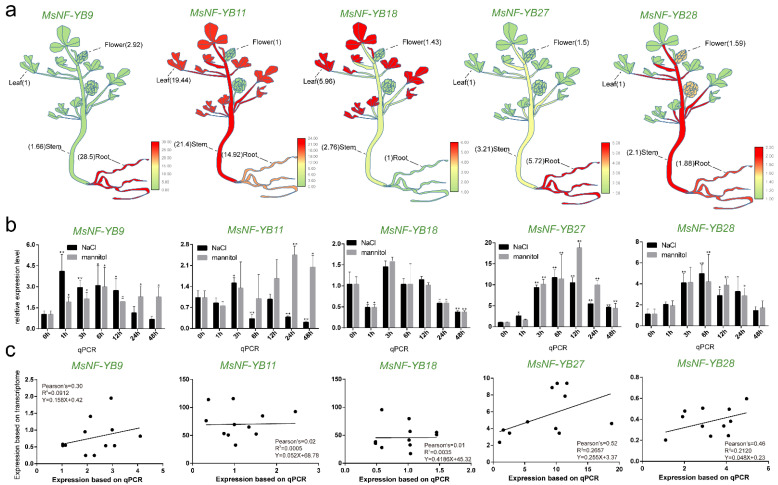
Quantification of gene expression levels of selected *NF-YB* genes from *M. sativa* using RT-qPCR. (**a**) Expression level of *MsNF-YB* genes in different tissues. (**b**) Expression level of *MsNF-YB* genes under NaCl and mannitol stress. Data are average of three independent biological samples ± SE, and vertical bars indicate standard deviation. ** indicates *p* < 0.01, and * indicates *p* < 0.05. (**c**) Correlation analysis of RT-qPCR and transcriptome (NaCl and mannitol) results for *MsNF-YB* genes. Pearson’s r indicates the Pearson correlation coefficient.

For *M. sativa*, the expression levels detected by RT-qPCR for *MsNF-YB9*, *11*, and *28* were relatively consistent with the transcriptome data (Figure 6a and Figure 9b): the expression level of *MsNF-YB9* was significantly higher in roots than in the other three tissues; *MsNF-YB11* was expressed at a relatively high level in all four tissues (Figure 9a and Supplementary file S5). Unlike transcriptome data, RT-qPCR data showed that *MsNF-YB18* had a significantly higher expression level in leaves than in other tissues. Meanwhile, RT-qPCR data showed that MsNF-YB27 was significantly expressed in roots, while transcriptome data showed its expression was the highest in pre-elongated stems (Figure 9a). Since the treatment timepoints for RT-qPCRs were the same as for the transcriptome data, we compared the transcriptome data and RT-qPCR data with the correlation analysis. RT-qPCR data showed that *MsNF-YB9*, *27,* and *28* showed a significant increase in gene expression under both NaCl and mannitol treatment (Figure 9b and Supplementary file S5), which was the same as for transcriptome data, and they were positively correlated (Figure 9c). Notably, *MsNF-YB9*/*MtNF-YB7*, *MsNF-YB27*/*MtNF-YB15,* and *MsNF-YB28*/*MtNF-YB16* were homologous gene pairs, and their expression was consistent under the two stress treatments, indicating that these genes play the same role in *M. truncatula* and *M. sativa*. Moreover, the expression level of *MsNF-YB11* was transiently elevated under NaCl treatment and then reduced greatly, which was inconsistent with its significant elevation as shown by the transcriptome data. The expression of *MsNF-YB18* detected by RT-qPCR was consistent with the changes of the transcriptome data (Figure 9c).

## 3. Discussion

Previous studies have demonstrated that NF-YB proteins play an important role in plant resistance to abiotic stresses [13]. The function of NF-YB in abiotic resistance was previously analyzed in several plant species, including *A. thaliana*, rice, wheat, tung tree, soybean, canola, grape, and tomato [9]. However, a genome-wide identification and characterization of *NF-YB* genes in *Medicago* is still lacking. In the present study, we conducted an integrated investigation on the *NF-YBs*, and a total of 21 *NF-YB* members from *M*. *truncatula* and 28 *NF-YB* members from *M*. *sativa* were identified.

Multiple sequence alignment confirmed that almost all *M. truncatula* and *M. sativa* NF-YB members contained DNA binding as well as NF-YA interaction and NF-YC interaction domains (Figure 1), and they shared highly conserved amino acid length, which was consistent with model plants such as rice [37]. Phylogenetic analysis indicated that they are highly homologous to NF-YB proteins from *Arabidopsis* (Figure 2), but one particular sub-cluster E was only presented in *Medicago*, which may play a special role in evolution. The same sub-clusters are more closely related, and the type and position of their patterns are similar (Figure 2 and Figure 3). These findings suggest that these motifs may be involved in the functional diversity of NF-YB proteins. In analyzing gene structure, we found that many *NF-YB* genes in *Medicago* had only one exon and no introns (Figure 3), which is consistent with the findings for *Arabidopsis* and *Brassica napus*. [30]. Previous studies have postulated that an intron-rich gene would lose multiple introns simultaneously by retrotransposition, thereby producing intron-less ancestral genes [38]. Thus, several *NF-YB* genes in *Medicago* may experience the loss of multiple introns during gene family diversification. Genome-wide analyses have shown that the loss and gain of introns were extensive during the process of eukaryotic diversification.

Duplication and divergence play a critical role in the expansion and evolution of gene families [39]. We found two segmental duplications (*MtNF-YB8*/*MtNF-YB15*, *MtNF-YB9*/*MtNF-YB14*) among 21 *MtNF-YB* genes (Figure 4), while two segmental duplication events (*MsNF-YB4*/*MsNF-YB11*/*MsNF-YB15*) among 28 *MsNF-YBs* were found in *M. sativa*. The gain and loss of genes or the expansion or contraction of gene families is common following polyploidization [40]. Unlike *M*. *truncatula*, *M. sativa* has tandem duplicated gene pairs (*MsNF-YB1*/*MsNF-YB2*). Thus, the expansion of the *MsNF-YB* gene family could be an indication that *MsNF-YB* genes play roles in additional biological processes or have novel functions. Previous studies have demonstrated that *NF-YB* plays an important role in drought resistance [13]. In our studies, ARE (anaerobic induction), TGACG-motif (MeJA-responsive), and ABRE (abscisic acid-responsive) elements were found widely distributed in *NF-YB* genes, and all of these elements were involved in response to drought stress, suggesting that the *NF-YBs* of *Medicago* are increased in response to drought stress.

High-salinity or drought soil is the most serious abiotic stress [41]. It is urgent to improve the salinity and drought tolerance of alfalfa to increase yield. Genechip data and transcriptome data under NaCl and drought treatments, along with expression profiles in various tissues, suggested that the expressions of six homologous gene pairs were highly induced or drastically changed in *Medicago* (Figure 6 and Figure 7). Previous studies have reported that *NF-YB* genes are also involved in plant developmental processes, including embryogenesis, flowering time, etc. [42]. In the present study, we identified the tissue-specific expression patterns of six *NF-YB* genes in *Medicago* in a variety of tissues, and the results showed that *NF-YB* genes were expressed ubiquitously, with the exception of a few genes that are expressed in specific tissues: *MtNF*-*YB16* is specifically expressed in flowers (Figure 8a), and *MsNF*-*YB9* in roots (Figure 9a). This observation was consistent with previous studies for *NF-YB* genes in rice [43], suggesting that *NF-YB* genes are multifunctional and are involved in a wide range of biological processes [44]. Subsequently, the expression pattern of all six genes was verified under NaCl and mannitol treatments in *M. truncatula* and *M. sativa* by RT-qPCR analyses (Figure 8b and Figure 9b), and the results suggested that several genes are involved in NaCl and/or mannitol induction. Correspondingly, three gene pairs (*MsNF-YB9*/*MtNF-YB7*, *MsNF-YB27*/*MtNF-YB15,* and *MsNF-YB28*/*MtNF-YB16*) were significantly up-regulated under NaCl and mannitol treatments, and their expression patterns were the same in *M. truncatula* and *M. sativa*. This evidence indicated that these genes are likely key genes in response to abiotic stress.

In the phylogenetic analysis, *NF-YB* genes were divided into five branches in addition to the E branch, which includes *Arabidopsis*-specific *NF-YB* genes. Among them, *NF-YB1*, *NF-YB2*, *NF-YB3*, *NF-YB6*, and *NF-YB9* have been extensively studied in *Arabidopsis*. Previous studies have shown that *NF-YB1* not only regulates drought resistance but also interacts with CO (CONSTANS) to affect the transcript levels of two key integrators (FT and SOC1) in the flowering pathway, therefore adjusting flowering time [45]. Interestingly, *MtNF-YB18* clustered with *AtNF-YB1*, indicating that it may have similar functions as *AtNF-YB1*. Moreover, *MsNF-YB2*, *27* and *MtNF-YB11*, *15* were observed to cluster with *AtNF-YB2* (Figure 2), which has been reported to regulate the photoperiod-dependent flowering time [46]. However, in our study, the homologous gene pair *MtGRF15*/*MsGRF27* showed significant high expression under both salt and mannitol treatments (Figure 8b and Figure 9b), suggesting that this gene pair may also play a role in coping with abiotic stresses.

In *Arabidopsis*, LEAFY COTYLEDON1 (LEC1) is a central regulator that controls many different aspects of embryo development. Early in embryogenesis, LEC1 is required to maintain the fate of embryonic cells that constitute the suspensor and to specify the identity of cotyledons, the embryonic leaves [47]. While previous studies have shown that NF-YB proteins of *Arabidopsis* can be divided into two classes, LEC1-like (LEC1 or AtNF-YB9; LEC1-LIKE or AtNF-YB6) and non-LEC1-like. *NF-YB9*/*LEC1* was the first *NF-YB* gene identified and studied in *A. thaliana* and has been shown to be required for the embryonic maintenance of cell fate, where the ectopic expression of *NF-YB9* can induce somatic embryos from vegetative cells [16]. In addition, *NF-YB9* has also been shown to play an essential role in embryogenesis and seed maturation [48]. *LEC1* and *LEC1-LIKE* (*NF-YB6*) regulated embryo development by activating the expression of genes required for embryogenesis and cellular differentiation [16]. In the present study, *MsNF-YB28* was grouped with *AtNF-YB9*, while *MtNF*-*YB16* was grouped with *AtNF-YB6* (Figure 2), suggesting they may share similar functions.

Furthermore, the homologous gene pairs (*MtNF-YB16/MsNF-YB28*) were all highly expressed under NaCl and mannitol treatment as evidenced by RT-qPCR (Figure 8b and Figure 9b). Thus, the homologous pairs may be involved in regulating embryonic development, as well as in response to abiotic stress. Therefore, these two candidate homologous genes (*MtGRF15*/*MsGRF27* and *MtNF-YB16*/*MsNF-YB28*) were considered as key candidates for in-depth study of *NF-YB* genes in *Medicago.*

## 4. Materials and Methods

### 4.1. Identification of NF-YB Family Members in Medicago

Based on the genome database, the sequence of *NF-YB* genes of *M. truncatula* and *M. sativa* were downloaded from the genome websites https://figshare.com/articles/dataset/Medicago_sativa_genome_and_annotation_files/12623960 (accessed on 1 September 2021) and https://www.jcvi.org/research/medicago-truncatula-genome-database (accessed on 1 January 2020), respectively. Moreover, the NFYB (PF00808) model profiles downloaded from Pfam (https://pfam.xfam.org/) were employed to identify putative *NF-Y* genes from *M. truncatula* and *M. sativa*. A further BLASTP search with an E-value cutoff of e^−10^ was performed to sort NF-Y family members using the AtNF-YB [2] and OsNF-YB [37] amino acid sequences as queries, which were retrieved from TAIR (http://www.arabidopsis.org) (accessed on 11 September 2020) and RICEDATA (http://www.ricedata.cn/gene) (accessed on 11 September 2020), respectively. In order to ensure the correctness of the selected genes, output putative NF-YB protein sequences were submitted to InterProScan (https://www.ebi.ac.uk/interpro/search/sequence-search) (accessed on 12 September 2020), CDD (https://www.ncbi.nlm.nih.gov/Structure/bwrpsb/bwrpsb.cgi) (accessed on 12 September 2020), Pfam (https://pfam.xfam.org/) (accessed on 13 September 2020), and SMART (http://smart.embl-heidelberg.de/) (accessed on 13 September 2020). Finally, 21 *MtNF-YB* and 28 *MsNF*-*YB* genes were identified, and they were assigned based on their locations on chromosomes. Correspondingly, the molecular weight (MW) and isoelectric point (pI) of the deduced amino acid sequences were predicted with the Expert Protein Analysis System (ExPASy) on the proteomics server (http://www.expasy.ch/tools/protparam.html) (accessed on 15 September 2020). The subcellular localization was predicted using the Softberry Home Page (http://www.softberry.com/) (accessed on 16 September 2020).

### 4.2. Analyses of Sequence, Conserved Motif, and Structural Characterization

To exhibit the structural divergence of NF-YB proteins, the conserved motifs were performed with Multiple Em for Motif Elicitation (MEME) 5.0.2 online program (https://meme-suite.org/meme/tools/meme) (accessed on 25 September 2020) [49]. The following parameters were employed: the maximum number of motifs was 20, minimum motif width was 10 (aa), and maximum motif width was 200 (aa). Subsequently, sequence alignment analysis of NF-YB protein sequences was carried out using jalview (https://issues.jalview.org/secure/Dashboard.jspa) (accessed on 25 September 2020). The visualization of exon–intron positions and conserved motifs was performed through TBtools software (Guangzhou, China) [50].

### 4.3. Analyses of Phylogenetic Relationship

NF-YB proteins from five plant species (*M. truncatula*, *M. sativa*, *A. thaliana,* and *O. sativa*) were used in a multiple alignment in ClustalW [51]. Phylogenetic trees were constructed by the neighbor-joining method using the program MEGA-X with a bootstrap of 1000 replicates. Meanwhile, subfamily clustering on the phylogenetic tree was determined based on *Arabidopsis* member clustering. Subsequently, EvolView (https://evolgenius.info/evolview-v2/) (accessed on 29 September 2020) was used to view the phylogenetic tree.

### 4.4. Analysis of Chromosome Locations and Collinearity

The loci of *NF*-*YB* genes were obtained from the genome annotation data. TBtools was applied to map the chromosome locations for each gene. Next, these sequences were analyzed to identify collinearity blocks against the whole genome using MCSCAN [52]. Moreover, the intraspecific synteny relationship (*M*. *truncatula* and *M. sativa*) and interspecific synteny relationship (*M*. *truncatula*, *M*. *sativa*, *A. thaliana,* and *O. sativa*) were analyzed, and they were mapped to the chromosomes of *M. truncatula* and *M. sativa* using TBtools, respectively. Lastly, the synonymous (Ks) and nonsynonymous (Ka) substitution rates were estimated using TBtools [53].

### 4.5. Identification of Stress-Response Cis-Elements of the NF-YB Promoter

The promoter sequences (length, 2 kb) of NF-YBs were collected by TBtools, and the *cis*-elements in the promoters were predicted using PlantCARE (http://bioinformatics.psb.ugent.be/webtools/plantcare/html/) (accessed on 2 October 2020). The visualized models of *cis*-elements in the promoters were made with TBtools [54].

### 4.6. Analysis of Gene Expression Profile

Genechip data from roots and shoots and those under drought and salt stress conditions for *MtNF*-*YB* genes were downloaded from the *M*. *truncatula* Gene Expression Atlas. The expression levels of *MtNF-YB* genes in different tissues were also analyzed. Amazing HeatMap software was used to generate the heatmap [50]. The original transcriptome data from *M*. *sativa* under NaCl and mannitol treatments at 0 h, 1 h, 3 h, 6 h, 12 h, and 24 h (SRR7160314-15, 22-23, 25-49, 51-52, 56-57) were downloaded from the NCBI database [55]. Then, the data were converted to fastq files using the SRA-Toolkit v2.9 [56]. Raw reads were trimmed using Trimmomatic-0.39 [57]. Gene expression level was determined by mapping cleaned reads to the corresponding alfalfa reference genomes using the StringTie v2.1.3 package and the log2(FPKM) values [58].

### 4.7. Plant Materials and Treatments

*M*. *truncatula* (cv. Jemalong A17) and *M*. *sativa* (cv. Zhongmu No.1) plants used in this study were stored at the Institute of Animal Sciences of the Chinese Academy of Agricultural Sciences, Beijng, China. Roots, stems, leaves, and flowers of mature *M*. *truncatula* and *M*. *sativa* plants were collected separately for RNA extraction and RT-qPCR analysis. To investigate the expression pattern of *NF-YB* genes in response to NaCl and mannitol stress, seeds were germinated and transferred into the MS liquid medium (MS basal salts supplemented with 30 g/L sucrose), then kept in a growth chamber at 25 °C under a photoperiod using a 16/8 light/dark regime. When the third leaf was fully expanded, seedlings were transferred to fresh MS liquid medium supplied with 300 mM NaCl and 15% mannitol, respectively, and the whole plants were collected at 0 h, 1 h, 3 h, 6 h, 12 h, 24 h, and 48 h for each treatment, and plants without treatment were used as controls. The samples were frozen in liquid nitrogen and stored at −80 °C for subsequent analysis.

### 4.8. Gene Expression Analyses by RT-qPCR

Total RNAs were extracted using Eastep^®^ Super total RNA Extraction kit (Promega, Shanghai, China) according to the manufacturer’s instructions. First-strand cDNA synthesis was performed using Trans^®^ Script One-Step gDNA Removal and cDNA Synthesis SuperMix (TransGen Biotech, Beijing, China) per the manufacturer’s recommendations. RT-qPCRs were performed with a 2’RealStar Green Fast Mixture (GeneStar, Shanghai, China) on an ABI Q7 QuantStudioTM Real-Time PCR Detection System (Applied Biosystems, CA, USA). PCRs were performed with the following program: 94 °C for 30 s, followed by 40 cycles of 94 °C for 5 s and 60 °C for 34 s. Melting curve analysis was performed using ABI Q7 QuantStudio^TM^ Real-Time PCR Software with the RT-qPCR data. The transcript levels of each gene were determined by relative quantification using the 2^-ΔΔCt^ method and normalized with the actin gene as a reference. Data are the average of three independent biological samples ± SE, and vertical bars indicate standard deviation. Student’s *t* test was used to compare the difference between two treatments (*n* = 3, * *p* < 0.05, ** *p* < 0.01) [59]. The primer sequences used in this study are shown in Table S1.

## Figures and Tables

**Table 1:** Properties of the predicted NF-YB genes in *M. truncatula* and *M. sativa*.

Gene Name	TIGR Locus	Homologous Gene	PI	MW (kDa)	Protein Length	Subcellular Localization	Plant TFDB
*MtNF-YB1*	MtrunA17Chr1g0158951	*MsNF-YB4*	4.64	12.44	112	Nuclear	Medtr1g028480.1
*MtNF-YB2*	MtrunA17Chr1g0159271	*MsNF-YB1*	5.28	11.57	108	Nuclear	Medtr1g029070.1
*MtNF-YB3*	MtrunA17Chr1g0159291	*MsNF-YB2*	4.66	10.36	91	Nuclear	Medtr1g029100.1
*MtNF-YB4*	MtrunA17Chr1g0165041	*MsNF-YB4*	5.59	21.65	191	Nuclear	Medtr1g039040.1
*MtNF-YB5*	MtrunA17Chr1g0185911	*MsNF-YB6*	5.75	19.25	175	Nuclear	Medtr1g072790.2
*MtNF-YB6*	MtrunA17Chr1g0191981	*MsNF-YB7*	6.90	14.46	128	Nuclear	Medtr1g083070.1
*MtNF-YB7*	MtrunA17Chr1g0195851	*MsNF-YB9*	5.72	22.24	195	Nuclear	Medtr1g088860.1
*MtNF-YB8*	MtrunA17Chr2g0290491	*MsNF-YB10*	5.78	17.99	167	Nuclear	Medtr2g026710.1
*MtNF-YB9*	MtrunA17Chr2g0296321	*MsNF-YB26*	8.88	28.96	257	Nuclear	Medtr0392s0020.1
*MtNF-YB10*	MtrunA17Chr2g0307551	*MsNF-YB11*	4.63	17.43	157	Nuclear	Medtr2g056000.1
*MtNF-YB11*	MtrunA17Chr3g0102351	*MsNF-YB12*	6.21	20.11	191	Nuclear	Medtr3g058980.1
*MtNF-YB12*	MtrunA17Chr4g0026311	*MsNF-YB14*	5.25	15.54	141	Nuclear	Medtr4g052950.1
*MtNF-YB13*	MtrunA17Chr4g0062271	*MsNF-YB11*	4.80	17.48	157	Nuclear	Medtr4g112380.1
*MtNF-YB14*	MtrunA17Chr4g0067091	*MsNF-YB26*	6.31	24.94	218	Nuclear	Medtr4g119500.1
*MtNF-YB15*	MtrunA17Chr4g0076321	*MsNF-YB27*	5.36	15.33	137	Nuclear	Medtr4g133938.1
*MtNF-YB16*	MtrunA17Chr4g0076381	*MsNF-YB28*	6.26	26.78	241	Nuclear	Medtr4g133952.1
*MtNF-YB17*	MtrunA17Chr5g0446491	*MsNF-YB18*	5.96	19.98	185	Nuclear	Medtr5g095740.1
*MtNF-YB18*	MtrunA17Chr5g0446601	*MsNF-YB20*	4.57	21.16	192	Extracellular	Medtr5g095900.1
*MtNF-YB19*	MtrunA17Chr7g0263601	*MsNF-YB6*	6.10	18.77	172	Nuclear	Medtr7g100650.1
*MtNF-YB20*	MtrunA17Chr8g0382931	*MsNF-YB16*	5.80	22.55	204	Nuclear	Medtr8g091720.1
*MtNF-YB21*	MtrunA17Chr8g0384451	*MsNF-YB17*	6.04	24.96	215	Nuclear	Medtr8g093920.1
*MsNF-YB1*	MsG0180001010.01.T01	*MtNF-YB2*	5.25	18.87	173	Nuclear	—
*MsNF-YB2*	MsG0180001011.01.T01	*MtNF-YB3*	5.17	22.86	204	Extracellular	—
*MsNF-YB3*	MsG0180001031.01.T01	*MtNF-YB2*	5.09	20.95	192	Nuclear	—
*MsNF-YB4*	MsG0180001571.01.T01	*MtNF-YB4*	5.32	21.27	188	Nuclear	—
*MsNF-YB5*	MsG0180003209.01.T01	*MtNF-YB17*	4.47	10.76	99	Nuclear	—
*MsNF-YB6*	MsG0180004009.01.T01	*MtNF-YB5*	5.75	19.26	175	Nuclear	—
*MsNF-YB7*	MsG0180004588.01.T01	*MtNF-YB6*	6.55	10.8	97	Nuclear	—
*MsNF-YB8*	MsG0180004726.01.T01	*MtNF-YB14*	6.42	21.34	182	Nuclear	—
*MsNF-YB9*	MsG0180004882.01.T01	*MtNF-YB7*	5.51	21.18	186	Nuclear	—
*MsNF-YB10*	MsG0280007510.01.T01	*MtNF-YB8*	5.78	17.67	163	Nuclear	—
*MsNF-YB11*	MsG0280009235.01.T01	*MtNF-YB10*	6.02	26.5	235	Nuclear	—
*MsNF-YB12*	MsG0380014538.01.T01	*MtNF-YB11*	6.21	20.18	191	Nuclear	—
*MsNF-YB13*	MsG0380014546.01.T02	*MtNF-YB17*	6.16	22.05	194	Extracellular	—
*MsNF-YB14*	MsG0480020531.01.T01	*MtNF-YB12*	5.40	14.64	130	Extracellular	—
*MsNF-YB15*	MsG0480020532.01.T01	*MtNF-YB12*	7.67	15.19	135	Extracellular	—
*MsNF-YB16*	MsG0480023372.01.T01	*MtNF-YB20*	5.80	21.81	197	Nuclear	—
*MsNF-YB17*	MsG0480023477.01.T02	*MtNF-YB21*	6.14	26.18	226	Nuclear	—
*MsNF-YB18*	MsG0580029966.01.T01	*MtNF-YB17*	4.92	15.87	144	Nuclear	—
*MsNF-YB19*	MsG0580029976.01.T01	*MtNF-YB18*	4.51	20.54	188	Nuclear	—
*MsNF-YB20*	MsG0580029978.01.T01	*MtNF-YB18*	4.64	20.68	187	Nuclear	—
*MsNF-YB21*	MsG0680031203.01.T01	*MtNF-YB17*	8.66	88.39	80	Nuclear	—
*MsNF-YB22*	MsG0780040897.01.T01	*MtNF-YB19*	8.67	90.71	289	Extracellular	—
*MsNF-YB23*	MsG0880043949.01.T01	*MtNF-YB17*	9.34	8.52	78	Nuclear	—
*MsNF-YB24*	MsG0880046588.01.T01	*MtNF-YB13*	6.92	31.34	277	Extracellular	—
*MsNF-YB25*	MsG0880046644.01.T01	*MtNF-YB13*	4.70	13.72	124	Nuclear	—
*MsNF-YB26*	MsG0880047027.01.T01	*MtNF-YB14*	5.27	14.69	130	Nuclear	—
*MsNF-YB27*	MsG0880047742.01.T01	*MtNF-YB15*	5.35	15.28	137	Nuclear	—
*MsNF-YB28*	MsG0880047744.01.T01	*MtNF-YB16*	6.25	23.97	214	Nuclear	—

## Data Availability

All data in the present study are available in the public database as referred in the Material and Method part.

## Supplementary Materials

The following are available online at https://www.mdpi.com/article/10.3390/ijms23136906/s1.
