# Supplementary figures and images for: Identification and Characterization of Abiotic Stress–Responsive NF-YB Family Genes in Medicago
Source: Int J Mol Sci. 2022 Jun 21;23(13):6906. doi: 10.3390/ijms23136906 (PMC9266772; doi:10.3390/ijms23136906)

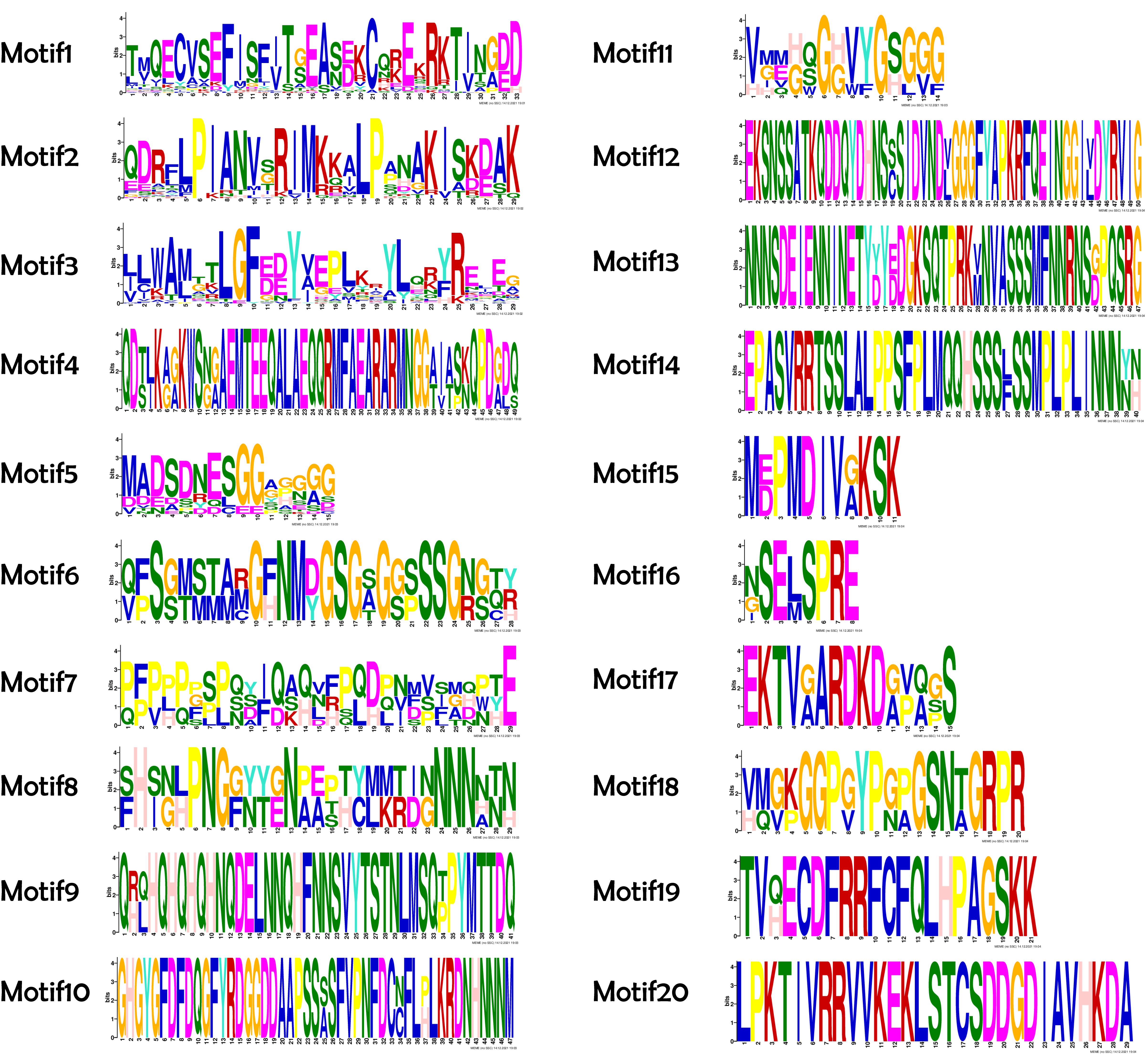

Supplement: Supplementary file 1 [file ijms-23-06906-s001.zip › ijms-1751900-supplementary/Supplementary files/NF-YB- Supplementary Figure/Supplementary Figure S1.tif]
